# Characterisation and mapping of a *Globodera pallida* resistance derived from the wild potato species *Solanum spegazzinii*

**DOI:** 10.1007/s00122-024-04605-0

**Published:** 2024-04-16

**Authors:** Ulrike Gartner, Miles R. Armstrong, Sanjeev K. Sharma, John T. Jones, Vivian C. Blok, Ingo Hein, Glenn J. Bryan

**Affiliations:** 1 Cell and Molecular Sciences Department, The James Hutton Institute, Invergowrie, Dundee, DD2 5DA UK; 2 School of Biology, University of St Andrews, St Andrews, KY16 9 UK; 3 School of Life Sciences, University of Dundee, Dundee, UK

## Abstract

**Key message:**

A new resistance locus acting against the potato cyst nematode *Globodera pallida* was mapped to chromosome VI in the diploid wild potato species *Solanum spegazzinii* CPC 7195.

**Abstract:**

The potato cyst nematodes (PCN) *Globodera pallida* and *Globodera rostochiensis* are economically important potato pests in almost all regions where potato is grown. One important management strategy involves deployment through introgression breeding into modern cultivars of new sources of naturally occurring resistance from wild potato species. We describe a new source of resistance to *G. pallida* from wild potato germplasm*.* The diploid species *Solanum spegazzinii* Bitter accession CPC 7195 shows resistance to *G. pallida* pathotypes Pa1 and Pa2/3. A cross and first backcross of *S.* *spegazzinii* with *Solanum* *tuberosum* Group Phureja cultivar Mayan Gold were performed, and the level of resistance to *G. pallida* Pa2/3 was determined in progeny clones. Bulk-segregant analysis (BSA) using generic mapping enrichment sequencing (GenSeq) and genotyping-by-sequencing were performed to identify single-nucleotide polymorphisms (SNPs) that are genetically linked to the resistance, using *S. tuberosum* Group Phureja clone DM1-3 516 R44 as a reference genome. These SNPs were converted into allele-specific PCR assays, and the resistance was mapped to an interval of roughly 118 kb on chromosome VI. This newly identified resistance, which we call Gpa VI^l^_spg_, can be used in future efforts to produce modern cultivars with enhanced and broad-spectrum resistances to the major pests and pathogens of potato.

**Supplementary Information:**

The online version contains supplementary material available at 10.1007/s00122-024-04605-0.

## Introduction

Potatoes are the world’s third most important staple food crop after rice and wheat (CIP 2017), with a worldwide production of 376 million tons in 2021 (FAO 2022). About 50% of potatoes are consumed fresh; the rest is mainly used for processing, starch production, and seed potato production (Birch et al. 2012). With the increasing demand for food from a growing human population of potentially 9.7 billion by 2050 (United Nations 2015), pest and disease management of potato crops are of increasing importance for food security. Environmentally benign methods of control, including natural resistance, are important for the sustainability of this crop.

Potato cyst nematodes (PCN) are highly specialised soil-borne biotrophic sedentary endoparasites of the Solanaceae family and the most economically important plant-parasitic nematode of potato (Gartner et al. 2021). The two species *G.* *rostochiensis* and *G. pallida* are the predominant PCN species globally with a third, *Globodera ellingtonae* (Handoo et al. 2012), being detected in the USA (Oregon and Idaho) (Skantar et al. 2011), and in the Andean region of South America (Lax et al. 2014). Turner and Subbotin 2013 estimated that about 9% of potato crop losses are due to infestations with PCN. PCN are quarantine pathogens in more than 100 countries worldwide (Mburu et al. 2020); therefore, containment procedures need to be applied when it is first detected (Pickup et al. 2018), putting additional costs on growers.

A primary strategy for the management of PCN is the breeding and use of potato cultivars that show resistance to one or more PCN species. The most commonly cultivated potato species *Solanum tuberosum* ssp. *tuberosum* does not show significant resistance to PCN. However, many related wild potato species and landraces show such resistances, which can, in many cases, be introgressed into commercial potato cultivars via conventional breeding. Genebanks all over the world maintain germplasm from wild potato species, landraces, and potato cultivars. Screening one such genebank, the Commonwealth Potato Collection (CPC), led to the discovery of the resistance *H1* to *G.* *rostochiensis* pathotypes Ro1 and Ro4 in *S.* *tuberosum* Group Andigena accession CPC 1673 (Ellenby 1952), which remains an important source of resistance against *G.* *rostochiensis*. Over 20 loci conferring resistance to PCN have been described in potato and the closely related tomato to date, and genes corresponding to three of these (*Gpa2*, *Hero*, *Gro1-4*) have been isolated (reviewed in Gartner et al. 2021). Most other resistances are conferred by one or more quantitative trait loci (QTL), carrying allelic variants which contribute to a usually incomplete resistance phenotype. All identified QTLs for PCN resistance are located at loci corresponding to the presence of resistance (*R*) gene clusters, which harbour variable numbers of genes encoding proteins belonging to the NB-LRR family, suggesting that such QTLs are also due to the action of NB-LRR genes (Gartner et al. 2021).

Following the publication of the first potato reference genome in 2011 (Potato Genome Sequencing Consortium 2011), the development of relatively inexpensive next-generation sequencing (NGS) approaches has led to new methods for linking phenotypes to specific single-nucleotide polymorphisms (SNPs), that can be used as markers for any phenotype of interest. These include novel high-throughput genome-wide genotyping approaches such as genotyping-by-sequencing (GBS) developed by (Elshire et al. 2011) and generic mapping enrichment sequencing (GenSeq) that targets single- or low-copy conserved genes dispersed throughout the potato genome (Chen et al. 2018). Genomic advances have also allowed the development of methods for the targeted mapping of plant resistance genes, notably resistance gene enrichment sequencing (RenSeq) (Jupe et al. 2013), a capture-based method which enriches for NB-LRR resistance gene sequences allowing the facile identification of sequence variants linked to the targeted resistance locus using bulked resistant and susceptible plants from a biparental cross. These methods generally require the availability of a reference genome. Several such genome assemblies such as the doubled monoploid DM clone (DM1-3 516 R44) of *S.* *tuberosum* Group Phureja (DM) (Potato Genome Sequencing Consortium 2011), the more recently published diploid inbred clone M6 derived from the wild potato species *S.* *chacoense* (Leisner et al. 2018) or the high-quality haplotype-resolved assembly of the autotetraploid potato cultivar Otava, as reported by (Sun et al. 2022), are available.

In this paper, we determine the chromosomal location and phenotypic characterisation of a novel *G. pallida* resistance locus that was detected in the diploid wild potato species *S.* *spegazzinii* accession CPC 7195. We deploy a variety of genetic approaches in our analysis, which will be informative for other studies aimed at mapping novel resistance loci in potato and other plant species.

## Material and methods

### Potato material

The diploid species *S.* *spegazzinii* is known to contain natural resistance to the PCN species *G. pallida* (Kreike et al. 1993, 1994). Accessions of *S. spegazzinii* from the CPC were screened for resistance to *G. pallida*, and accession CPC 7195 was identified as highly resistant. True seeds from this accession were germinated, grown to produce tubers, and one resistant plant (#10) was chosen for crossing to a susceptible genotype named Mayan Gold (also known as *S.* *tuberosum* Group Phureja DB337(37)), which is a diploid potato cultivar that is susceptible to both *G. pallida* and *G.* *rostochiensis,* and which produces long, yellow-fleshed tubers. 03.F1.3a(35), a progeny clone (#35) from a cross of *S.* *spegazzinii* accession CPC 7195(10) to Mayan Gold, was chosen as the PCN-resistant parent of a backcross population (comprising 993 clones) named 13.A.02, created by crossing 03.F1.3a(35) to Mayan Gold. *S.* *tuberosum* cv. Desirée is a tetraploid commercial potato cultivar. It is susceptible to PCN and used as a positive control for susceptibility in resistance assessment experiments.

### PCN populations

*G. pallida* populations Pa1 JHI (pathotype Pa1), Lindley JHI (pathotype Pa2/3, formerly Pa2) and Luffness JHI f1 (pathotype Pa2/3, formerly Pa3), as well as *G.* *rostochiensis* pathotype Ro1, were used in this study.

### PCN resistance assessments

Two bio-assays were used to determine the level of resistance to PCN in different potato genotypes. In the high-throughput but more variable root trainer bio-assay, potato plants were grown in PCN infested soil (15 cysts per plant) in four-chambered root trainers; then, the number of female nematodes visible on the surface of the “root-ball” was counted 7–8 weeks post-inoculation. In the more accurate but lower throughput pot bio-assays (Reid et al. 2015), newly grown cysts extracted from a sand/loam 1:1 mixture were counted at 12 weeks after inoculation with cysts. The cysts were bagged to ensure that they are not counted as new cysts after the experiment is completed. Both bio-assays were performed in four biological replicates. To rank the degree of resistance in the pot bio-assay, the number of cysts that developed in the different potato clones was converted into a standard resistance scoring notation (EPPO 2006). The relative multiplication rate of PCN with potato genotype Desirée as a reference was determined and according to EPPO 2006 categorised into nine levels of resistance (1–9), with 1 being the lowest and 9 the highest level of resistance.

### Heritability of cyst count trait in 13.A.02 population

A general analysis of variance (ANOVA) was performed for the cyst count data using the mean cyst count phenotype for each progeny clone and replicate data (four replicates per clone). Genotypic and residual mean square (MS) values from the ANOVA were used to calculate the overall genotypic variance (*σ*_g_^2^), and the environmental variance (*σ*_e_^2^), broad-sense heritability (*H*^2^) of the cyst count data. The broad-sense heritability (*H*^2^) of clone means was estimated as follows**:**

$$H^{2} = \sigma_{g}^{2} /(\sigma_{g}^{2} + (\sigma_{e} ^{2} /4))$$

where ***σ***_***g***_^***2***^ = (Genotypic MS – Residual MS)/4 (4 represents the number of reps) and ***σ***_***e***_^***2***^ = Residual MS.

### Extraction and quantification of plant genomic DNA

Genomic DNA was extracted from young leaf tissue from individual glasshouse grown plants including parents using either the Qiagen mini kit, Qiagen DNeasy Plant Maxi Kit or QIAamp 96 DNA QIAcube HT following the manufacturer’s protocol and quantified using the Quant-iTTM PicoGreen® dsDNA Assay Kit (Invitrogen, San Diego, CA).

### DNA purification procedures

#### Purification of gDNA with magnetic beads

AMPure XP beads from Beckman Coulter were used according to the manufacturer’s protocol.

#### Purification of PCR products for sequencing

The ExoSAP-IT™ kit from ThermoFisher was used to enzymatically remove unused primer and dNTPs from PCR reactions, which would otherwise interfere with DNA sequencing reactions. The kit was used according to the manufacturer’s protocol.

#### Purification of PCR products for GBS

The Qiagen QIAquick PCR purification kit (Qiagen cat. No. 28106) was used according to the manufacturer’s recommendations.

### Genotyping methods

Two main genotyping methods were deployed in this study, GenSeq and GBS. GenSeq was applied and analysed initially to map the resistance; GBS was performed with the aim to get more SNPs that are linked to the resistance and to facilitate fine mapping of the resistance locus. The two methods are both genome complexity reduction-based approaches. We essentially used the methods published for GenSeq in (Chen et al. 2018) and for GBS in (Poland et al. 2012) with modifications to the restriction enzymes used for library construction. GBS libraries were constructed using double (*Pst*I*-Mse*I) restriction enzyme digestion and sequencing was performed on Illumina HiSeq 4 k platform to generate 150 bp paired-end sequence reads.

For both GenSeq and GBS, the sequencing entailed the isolation of genomic DNA from parents and progeny of the crosses used. In both cases libraries were constructed from resistant parent, susceptible parent, resistant bulk, and susceptible bulk. The two phenotypic bulks were comprised of 20 equimolar samples from individual plants possessing or lacking the resistance trait, respectively. All DNA libraries were barcoded (indexed) at the individual plant level. For GenSeq, the enrichment for target sequences was performed by hybridisation (capturing) with an RNA bait library corresponding to single- or low-copy genes, as described by Chen et al. 2018, the sequences of the probe library for GenSeq can be accessed at https://ics.hutton.ac.uk/solanum/.

#### GenSeq

Adapters were trimmed with the software Fastq-mcf (Aronesty 2011) (v1.04.676). Trimmed reads were then mapped onto the DM potato reference genome version 4.03 (Potato Genome Sequencing Consortium et al. 2011; Sharma et al. 2013) using Bowtie2 (Langmead and Salzberg 2012) (v2.0.6, default mode for multi-mapping) program. The resulting BAM files for both the parents, and the bulks were merged and indexed using the software package SAMtools v0.1.18, with SAMtool mpileup (Danecek et al. 2021). The pileup files were generated for both bulks and parents and the software VarScan v2.3.7 (Koboldt et al. 2012) was used for variant calling.

#### GBS

Sequence reads from GBS libraries were demultiplexed using GBS-SNP-CROP-v.4.1 (Melo et al. 2016), quality trimmed using Trimmomatic (Bolger et al. 2014) and mapped onto the DM potato reference genome version 4.03 (Potato Genome Sequencing Consortium 2011; Sharma et al. 2013) using Bowtie2 (Langmead and Salzberg 2012). Further bioinformatic processing and variant discovery (using HaplotypeCaller) was performed using GATK tools (McKenna et al. 2010; DePristo et al. 2011).

#### KASP assay design and validation

Putative informative SNPs on chromosome VI obtained by GenSeq (Fig. 4c) and the SNPs obtained by GBS on chromosome VI were used to design KASP assays, containing two allele-specific forward primers and one common reverse primer (Supplementary Table S1) using the annotated SNP sequences. DNA samples from parental, bulk and recombinant plants were subjected to genotyping with the KASPassays (LGC Biosearch Technologies, Middlesex, UK).

## Results

### *S. spegazzinii*-derived PCN resistance protects against various PCN populations

**Fig. 1:**
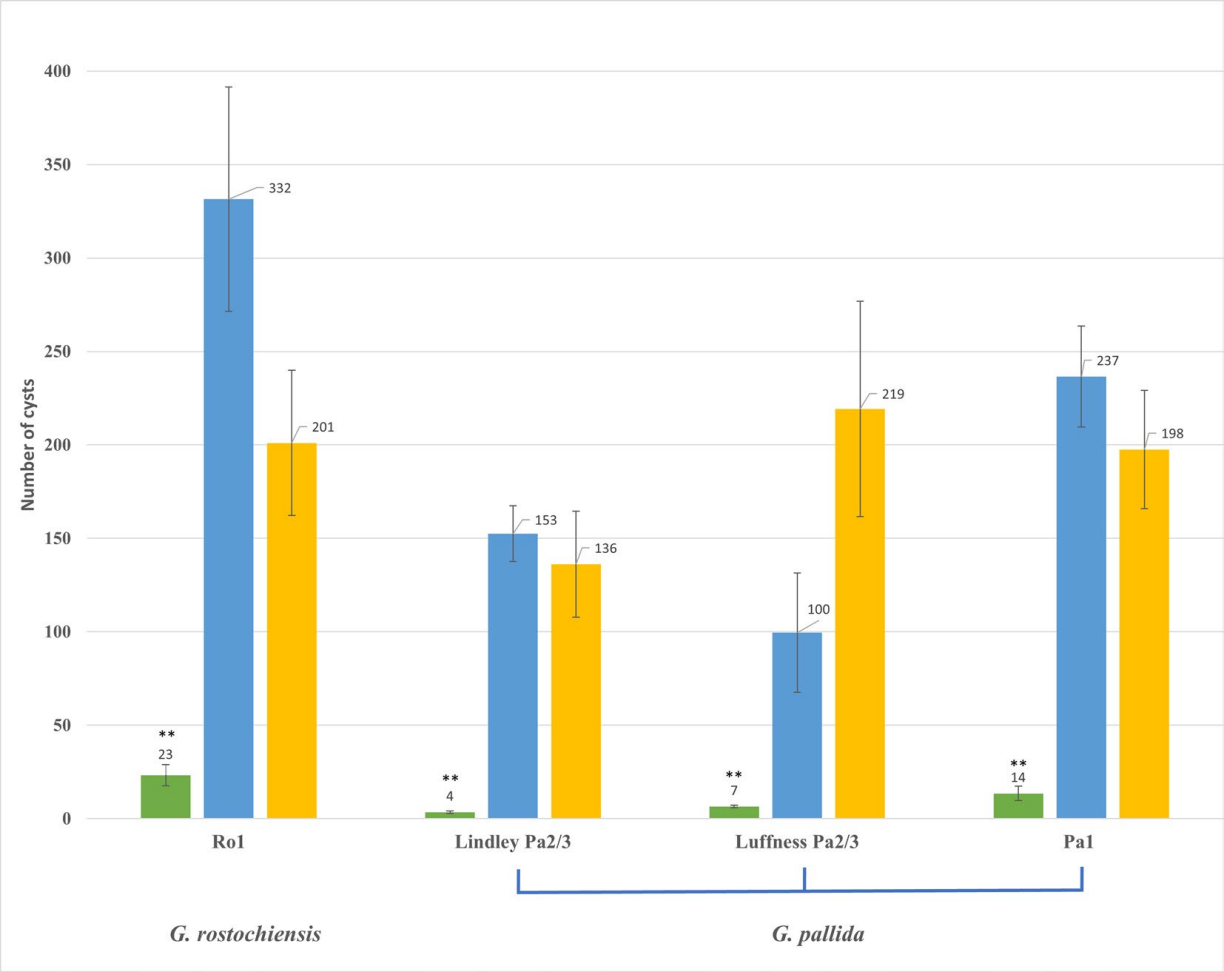
Resistance to different PCN populations in 03.F1.3a(35), DB337(37) and Desirée. Using a pot-based bio-assay, the level of resistance for different PCN populations was determined for the parental lines 03.F1.3a(35) (green bars) and DB337(37) (blue bars), with Desirée (yellow bars) serving as a susceptible control. The number of cysts is highly reduced in the resistant parent for all four PCN populations tested. The cyst numbers for the susceptible parent DB337(37) are comparable to the numbers obtained for Desirée. ** indicates that the number of nematodes is highly significantly (*p* < 0.01) lower than on Desirée and DB337(37)

The level of PCN resistance in *S.* *spegazzinii* to three UK *G. pallida* populations, “Lindley” Pa2/3, “Luffness” Pa2/3 and Pa1, which correspond to the three introductions to the UK and that differ in their virulence against other resistance sources, was assessed. In addition, resistance to *G.* *rostochiensis* Ro1 was assessed in the parental lines 03.F.1.3a(35) and DB337(37) by pot-based bio-assays. The potato cultivar Desirée served as a positive control for PCN susceptibility. The original P_0_ clone *S.* *spegazzinii* CPC 7195(10) had been lost prior to the start of this project due to a failure to produce the tubers necessary for further clonal propagation. The resistant F1 parent 03.F.1.3a(35) showed a reduced number of cysts across all four PCN populations tested, ranging from 4 to 23 cysts per pot at 12 weeks post-inoculation (Fig. 1, green bars) and was used as a control for resistance. In contrast, the susceptible parent DB337(37) showed cyst numbers that are comparable to those observed for the susceptible Desirée control, ranging from 100 to 332 cysts (Fig. 1, blue and yellow bars, respectively). The differences between the resistant parent and the two susceptible lines were statistically significant, as determined using Fisher’s exact test. Table 1 shows the degree of resistance of the different PCN populations in the parental lines 03.F.1.3a(35) and DB337(37) as standard resistance scoring notation (EPPO 2006). In the susceptible parent DB337(37) all tested PCN populations can develop similar to the reference potato cultivar Desirée. The *S.* *spegazzinii*-derived resistant parent 03.F.1.3a(35), on the other hand, shows different degrees of resistance to PCN populations. The development of the *G. pallida* populations Lindley, Luffness and Pa1 is highly inhibited, with a multiplication rate of less than 3% of the reference. The resistance of these potato genotypes to *G.* *rostochiensis* pathotype Ro1 is not as strong as for the tested *G. pallida* populations, however it shows moderate resistance with a cyst multiplication rate of less than 10% of the reference (Table 1). These results also demonstrate that mapping the resistance locus or resistance loci using progeny from a cross between resistant 03.F.1.3a(35) and susceptible DB337(37) should be feasible with an appropriate segregating population. Given that 03.F.1.3a(35) results from a cross between the original *S.* *spegazzinii* accession and highly susceptible DB337(37) any resistance factors present in 03.F.1.3a(35) should be heterozygous and segregate in a population derived from these two genotypes.

**Table 1:** Level of resistance in different PCN populations

	PCN population			
Potato genotype	*G. rostochiensi*	*sG. pallida*		
	A Ro1	Lindley	Luffness	
		Pa2/3	Pa2/3	Pa1
Desirée	2	2	2	2
03.F1.3a(35)	6	8	8	9
DB337(37)	1	2	3	1

The cyst multiplication rate relative to Desirée as reference was determined and converted to the resistance scoring factor. To get the standard resistance scoring, the relative multiplication rate is categorised into nine levels, 1 being very susceptible (> 100% of the multiplication rate of Desirée) and 9 being the highest level of resistance (less than 1% of the multiplication rate of Desirée) (EPPO 2006)

### Segregation pattern of the resistance to *G. pallida* in the 13.A.02 population

**Fig. 2:**
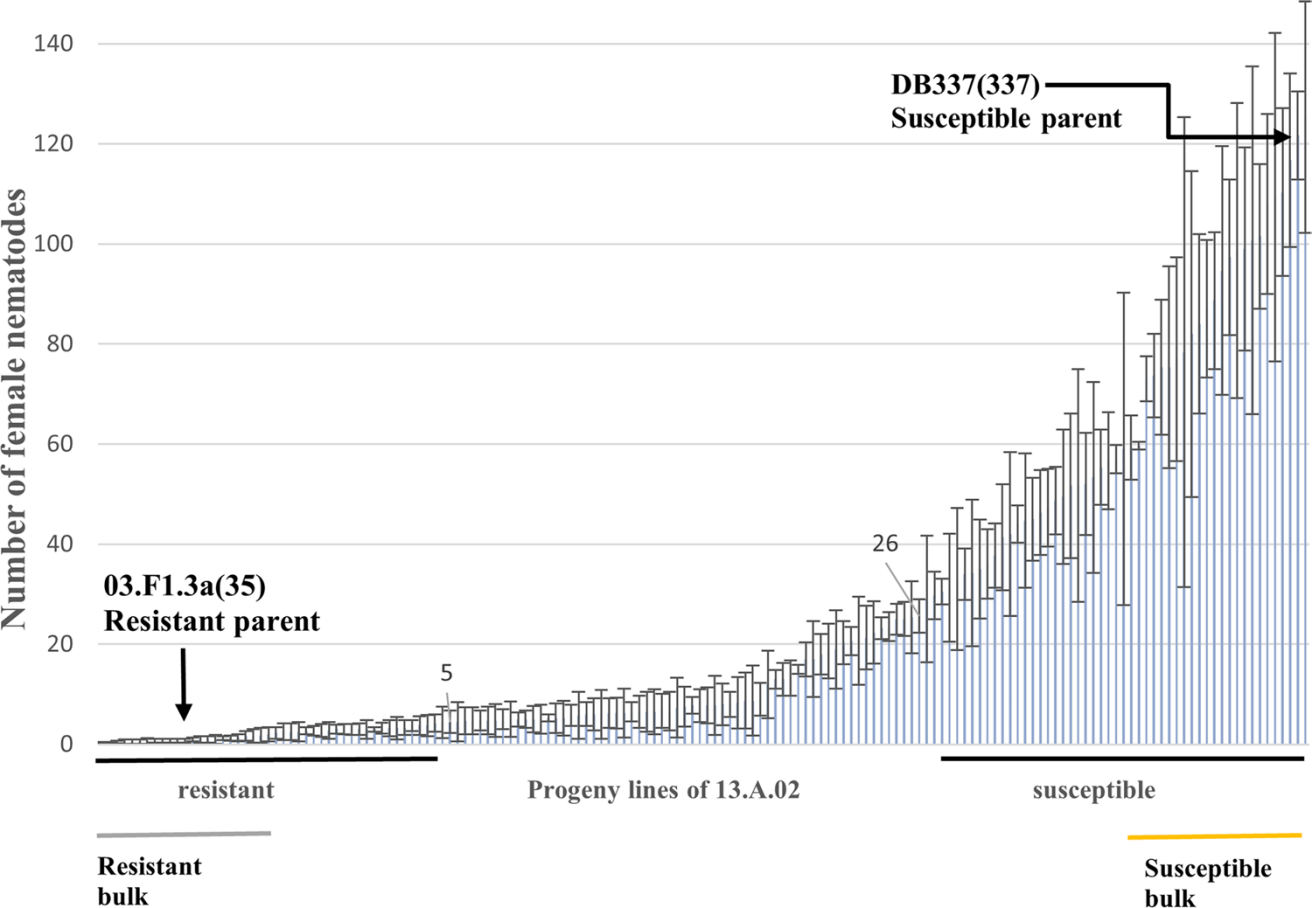
Determination of *G. pallida* resistant and susceptible progeny clones of 13.A.02. This diagram shows the average number of female nematodes in the 158 initially tested progeny clones of 13.A.02 in a root trainer assay. Sixty clones showed a high level of resistance with a maximum of 5 female nematodes on the outside roots, 46 showed intermediate susceptibility/resistance, and 52 were susceptible. The resistant and susceptible parents are indicated by black arrows

Progeny clones from the backcross population 13.A.02 were used to map the resistance locus or loci. Initially, a random subset of 200 of the 993 backcross clones were selected and the segregation pattern of the resistance and susceptibility to *G. pallida* “Lindley” Pa2/3 was determined using root trainer assays. In 158 of 200 cases, backcross progeny clones could be propagated in three or four replicates and individually assessed for resistance. The segregation patterns of *G. pallida* Lindley Pa2/3 resistance are shown in Fig. 2 together with the resistance scores of the parental lines. The resistant 03.F.1.3a(35) line showed an average of 1 female nematode per root trainer, while the susceptible DB337(37) scored an average of 122 female nematodes. To be scored as fully resistant, a maximum of 5 female nematodes were allowed to grow on the surface roots of the root trainer. On the other hand, if a plant growing in a root trainer showed more than 25 female nematodes on the surface roots, it was defined as fully susceptible. Using these criteria, the 158 plants segregated into 51 fully susceptible and 61 fully resistant clones. In addition, 46 clones showed intermediate responses with an average count of 6 to 24 female nematodes on the surface. The numbers of clones with a high level of resistance and susceptible clones conform very closely to the 1:1 ratio expected from the segregation of a single dominant gene. The observed reduced level of susceptibility/partial resistance could be a consequence of a weak resistance allele or a favourable combination of susceptibility alleles that somewhat restrict PCN establishment (for a review about susceptibility alleles see Koseoglou et al. 2022). Comparing the expectations from diploid Mendelian genetics with the observed phenotypes of the backcross progeny suggested that either a single major heterozygous dominant gene or one major and one minor gene present in the resistant parent are likely to be responsible for the pattern of resistance observed. The observed level of resistance in the backcross progeny is primarily due to genetic factors, as shown by the high broad-sense heritability of 0.87 (Clone MS = 3209.45, 157 df, Residual MS = 408.06, 399 df, *F* = 2.43, *P* < 0.00001).

### The *S. spegazzinii* resistances to *G. rostochiensis* and *G. pallida* do not co-segregate

**Fig. 3:**
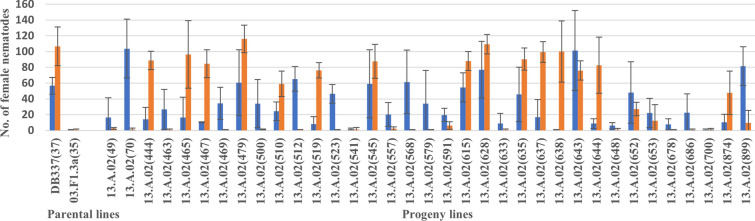
The resistances to *G. rostochiensis* and *G. pallida* do not co-segregate. Root trainer assays for assessing the segregation pattern for *G.* *rostochiensis* and *G. pallida* in the 13.A.02 line were performed, and the results compared. The blue bars indicate *G.* *rostochiensis* and the orange bars *G. pallida* infection levels. The numbers of females are plotted for each potato line tested; the parents serve as controls. The infection assays were repeated four times per clone

We next examined whether the resistance to *G. pallida* Lindley Pa2/3 and to *G.* *rostochiensis* Ro1 are conferred by the same resistance locus. If the resistances are conferred by the same locus, the plants that have been identified as either resistant or susceptible to *G. pallida* Lindley Pa2/3 would also be resistant or susceptible, respectively to *G.* *rostochiensis*. Root trainer assays were performed on 35 clonal lines that were classified as fully resistant (17) or susceptible (18) to *G. pallida,* to compare resistance/susceptibility of *G. pallida* to *G.* *rostochiensis,* including the parental potato lines. An analysis of the number of female nematodes developing on the surface roots confirmed the previous phenotypic assessment of resistance against *G. pallida* Lindley Pa2/3. However, the lack of correspondence between female nematode counts for *G. pallida* Lindley Pa2/3 and *G.* *rostochiensis* indicates clearly that the resistance *loci* do not co-segregate (Fig. 3).

These data are consistent with the resistance against Lindley Pa2/3 and *G.* *rostochiensis* Ro1 being due to genes acting at different loci.

### Identification of informative SNPs linked to the *G. pallida* resistance locus using GenSeq

For the genetic mapping of the resistance locus using a bulk-segregant analysis (BSA) approach, the extreme phenotypes with the 20 most and 20 least resistant clonal lines were selected and used as bulks for the enrichment sequencing. These lines, together with the number of female nematodes scored in the root trainer assay, are listed in Supplementary Table S2.

**Fig. 4:**
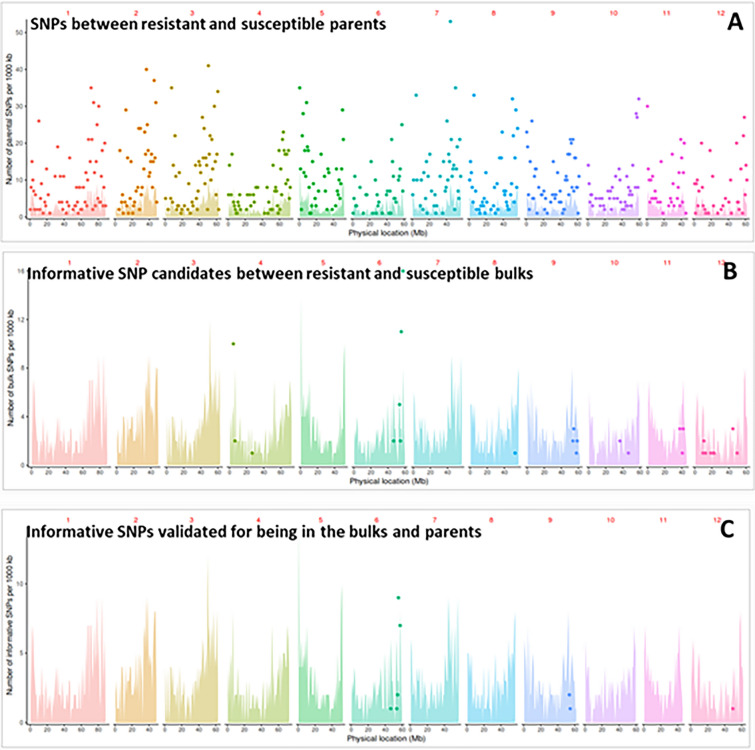
Distribution of SNPs identified by GenSeq at 5% mismatch rate. Each set of coloured data represents a specific chromosome. Coloured “spikes” represent the number of single and low copy number genes targeted by probes across each chromosome. Each dot represents SNPs, and its placement on the *y*-axis determines the number of SNPs identified in a 1 Mb bin. Panel A shows the SNPs identified between the two parental lines. Panel B shows the SNPs found in the two bulks with the expected ratio of the alternative allele being 0.5 ± 0.1 for the resistant bulk and 0 or 1 ± 0.1 for the susceptible bulk. Panel C shows the informative SNPS identified and validated to be intragenic in the bulks and parents

GenSeq was used as a tool to identify informative SNPs that can be used for mapping the resistance to *G. pallida*. Indexed GenSeq enrichment libraries were constructed, and sequence reads were mapped onto the DM reference genome. Supplementary Table S3 shows the total number of GenSeq reads and the number mapped to the reference genome at different mismatch rates. The on-target rate ranged from 52 to 89%, depending on the mismatch rate used. In Supplementary Table S4, the number of SNPs determined in bulks, parents and both are listed for different mismatch rates. The results of the GenSeq analysis at a 5% mismatch rate are illustrated in Fig. 4. At this mismatch rate, 4637 parental SNPs (Fig. 4, Panel A) are present, 77 SNPs from the bulks passed the filtering for being heterozygous (i.e. observed at a ratio of 40–60% for the resistant or susceptible allele) in the resistant bulk and being homozygous (i.e. ratio of 0–10% for the resistant allele and 90–100% for the susceptible allele) in the susceptible bulk. (Fig. 4, Panel B). *S. spegazzinii* and *S. tuberosum* group Phureja are not very closely related potato species; therefore, the number of SNPs between the parents is expected to be much higher than between the bulks. In the bulks, the parental SNPs across the genomes have been randomised with the exception of the resistance interval which has been fixed based on the phenotypic observations. Figure 4, Panel C shows the 24 SNPs that remained, when they were validated for having the expected frequency in both parents and bulks and being intragenic.

In total, 20 informative SNPs were identified on chromosome VI, three on chromosome IX, and one on chromosome XII. Their gene IDs and the number of SNPs in each gene are shown in Supplementary Table S5.

### KASP assays to confirm linkage between chromosome VI markers and resistance locus

**Fig. 5:**
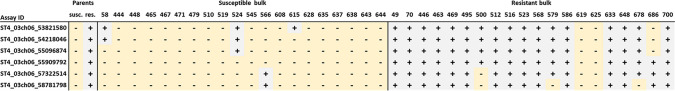
Graphical genotyping of the individuals of the bulks (KASP assays from GenSeq). The KASP assays are sorted by chromosome position; ST4_03ch0653821580 is upstream of ST4_03ch0658781798. The clones labelled – in light yellow have the allele of the susceptible parent in the SNP tested; the clones labelled + in light grey share the alleles from the resistant parent

KASP primers were generated from six informative SNPs on chromosome VI between positions 53,821,580 and 58,781,798 bp obtained from the GenSeq analysis with DM as reference and are listed in Supplementary Table S1. KASP marker assays were performed on each individual plant of the resistant and susceptible bulks used for the enrichment sequencing. Figure 5 shows the results, presented as graphical genotypes.

The GenSeq-derived KASP markers from chromosome VI are linked to the phenotypes of the parents and the phenotypic bulks; eight of the 40 plants were identified as being recombinant within the region defined by the six KASP markers, suggesting that the resistance locus resides on chromosome VI in an interval defined by SNP markers ST4_03ch06_55096874 and ST4_03ch06_57322514. One of the recombinant plants, susceptible clone 566 from the bulk, was lost, so it could not be tested with additional KASP assays.

Two previously identified resistant individuals (619 and 625) do not fit this general pattern and show marker genotypes consistent with being susceptible. Consequently, clones 619 and 625 were retested for the level of resistance in a pot test, which is more sensitive than a root trainer assay, and found not to be fully resistant, but to have reduced susceptibility with an average cyst count of 60 (± 9) and 50 (± 34), respectively, with a value of 216 (± 122) for the susceptible parent and 13 (± 9) for the resistant parent.

### Identifying additional informative SNPs in the region of interest using GBS

The GenSeq analysis did not provide any additional informative SNPs within the resistance locus on chromosome VI between DM positions 55,909,792 to 57,322,514 bp, an interval of approximately 1.42 Mb. To further narrow down the region containing the resistance locus, GBS, performed with the individually labelled DNA of the resistant and susceptible clones used as bulks in GenSeq of the cross 13.A.02 except clones 13.A.02(619) and 13.A.02(625), and their parents were used. The sequences were visualised in the software Tablet 1.19.09.03 (Milne et al. 2013). Reads in the region of the identified PCN resistance locus on chromosome VI from position 55,909,792 to 57,322,514 bp with a reading depth of at least 50 were screened for having two alleles in the ratio 0.4 to 0.6 (*i.e*., approximately 1:1) in 03.F1.3a(35) and then checked to determine whether the corresponding position in DB337(37) had only one allele. If that was the case, the SNP pattern in the progeny clones was determined. Supplementary Table S1 shows the SNPs found to be linked to resistance/susceptibility that were used in KASP or sequencing assays.

### Use of recombinants to further localise the *G. pallida* resistance locus in *S. spegazzinii* CPC 7195

**Fig. 6:**
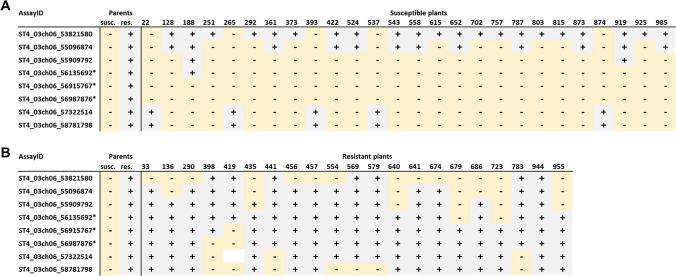
Graphical genotyping with a recombination event near the resistance locus. The clones shown represent the progeny of the cross 13.A.02 that had a recombination event near the resistance interval and showed a clear phenotype. The clones labelled – in light yellow have the allele of the susceptible parent in the SNP tested; the clones labelled + in light grey share the alleles from the resistant parent. The white cells indicate that the assay did not work. The KASP assay labelled with an * was obtained by SNPs obtained by GBS. Panel A shows the allele patterns from the parents and the susceptible bulk, Panel B from the parents and the resistant bulk.

To further refine the position of the resistance locus on chromosome VI, additional resistant and susceptible backcross clones with a recombination event between positions 55,096,874 and 57,322,514 were needed. All 993 progeny clones of 13.A.02 were therefore screened for recombinants using the two KASP markers (ST4_03ch06_55096874 and ST4_03ch06_57322514) that flank the resistance locus with DNA obtained from the original clones. Of the 882 clones that yielded robust KASP data in both assays, 112 were recombinant in the target region on chromosome VI. These recombinant clones were phenotyped in a root trainer assay and the same plants used for the root trainer assay were tested again with the KASP assays, to both eliminate tubers that were accidentally mislabelled during the years of maintenance and to determine which plants scored with either fewer than 6 (resistant) or more than 25 female (susceptible) nematodes in the root trainer assay. In total, 21 resistant clones and 26 susceptible clones were chosen for further fine mapping. The high number of plants with intermediate phenotype and the high variation in nematode numbers in susceptible plants within replicates might partly be due to a combination of susceptibility alleles that restrict PCN reproduction, environmental conditions and partly reflect the limits of the root trainer assay. These recombinants were tested with additional KASP markers derived from GBS corresponding to positions 53,821,580 to 58,781,798 on chromosome VI. Figure 6 shows the graphical genotyping results from the recombinant clones tested. The results of the other marker assays are shown to test for consistency. The susceptible clone 188 in Fig. 6a shows the “resistance pattern” only upstream from position 56,135,692, indicating that the resistance locus must therefore be “downstream” of this position. Figure 6b shows resistant clone 419 with the “susceptibility pattern” downstream from position 56,915,767; therefore, the resistance locus must be “upstream” of this position, and finally, clones 679 and 723 show the “susceptibility pattern” upstream from position 56,135,692; therefore, the resistance locus must be downstream from this marker position.

In summary, by using KASP assays designed from SNPs obtained by GenSeq and GBS analysis to analyse an additional 52 recombinant backcross progeny plants resulted in the delineation of the resistance locus to a region of 780 kb on chromosome VI between position 56,135,692 and 56,915,767, with three resistant (419, 679 and 723) and one susceptible (188) recombinant clones remaining for further analysis.

**Fig. 7:**
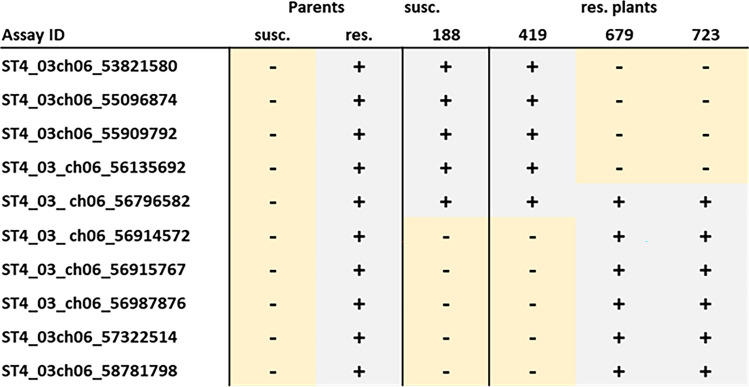
Further narrowing down the resistance by graphical genotyping. SNP candidates obtained from GBS (labelled with *) and KASP assays were used. The clones labelled – in light yellow have the allele of the susceptible parent in the SNP tested, and the clones labelled + in light grey share the alleles from the alleles from the resistant parent

To further narrow down the position of the resistance locus, these four recombinant plants (188, 419, 679 and 723) and parents were genotyped by Sanger sequencing the PCR product in the region of the informative SNPs on chromosome VI positions 56,796,582 and 56,914,572 detected by GBS. Figure 7 shows the graphical genotypes for all KASP and sequencing assays performed. The graphical genotypes for susceptible clone 188 and resistant clone 419 suggest that the resistance is located within the interval of chromosome VI positions 56,795,582 and 56,9145,572 that corresponds to 118 kb in the DM reference genome, a region which, according to SpudDB,^1^ harbours 14 high-confidence gene models (Table 2).

**Table 2:** List of genes in DM in the interval with the resistance to *G. pallida* in *S. spegazzinii CPC 7195*

Gene name	Description	Position on chr.VI
Soltu.DM.06G032060.1	Laccase/diphenol oxidase family protein	56,797,112..56800721
Soltu.DM.06G032070.1	Homeodomain-like transcriptional regulator	56,816,128..56818397
Soltu.DM.06G032080.1	Glycosyl hydrolase family protein	56,820,344..56824583
Soltu.DM.06G032090.1	Josephin protein-related	56,828,401..56829147
Soltu.DM.06G032100.1	Thylakoid soluble phosphoprotein TSP9 domain-containing protein	56,833,232..56834013
Soltu.DM.06G032110.1	Cytochrome P450, family 86, subfamily A, polypeptide	56,836,408..56838552
Soltu.DM.06G032120.1	Zinc finger (C2H2 type) family protein	56,849,541..56851152
Soltu.DM.06G032130.1	Golgi nucleotide sugar transporter	56,854,375..56859689
Soltu.DM.06G032140.1	Myb-domain protein	56,862,756..56865014
Soltu.DM.06G032150.1&.2	Tetratricopeptide repeat (TPR)-like superfamily protein	56,873,096..56876786
Soltu.DM.06G032160.1	Cell division control, Cdc6	56,895,973..56902969
Soltu.DM.06G032170.1	Ribosomal protein L10 family protein	56,903,608..56905903
Soltu.DM.06G032180.1&.2	Quinone reductase family protein	56,908,586..56912059
Soltu.DM.06G032190.1	Pleckstrin homology domain-containing protein 1	56,913,478..56914487

These are the high-confidence genes detected in DM (v6.1) in the chromosome VI region 56,795,582 to 56,9145,57

## Discussion

### A potentially useful new source of potato resistance to PCN species *G. pallida*

A powerful approach for managing PCN, ideally as part of integrated pest management (IPM), is the use of resistant potato varieties. Many wild potato species and landraces, often native to Mexico and the Andean Highlands, possess resistances to pathogens, including PCN. The most commonly cultivated potato species in Europe, *Solanum tuberosum* ssp. *tuberosum,* originally lacked this genetic diversity and consequently did not show resistance to the major pathogens. This was demonstrated most poignantly during the potato famine in the middle of the nineteenth century, and as a consequence of this, potato breeding for resistance to the main pests and pathogens became a priority. With the development of molecular markers, it has become possible to identify different sources of *G. pallida* resistance and to introgress them into breeding clones and cultivars (*e.g.* potato variety Buster which shows resistance to both *G. pallida* and *G.* *rostochiensis* (Griffin et al. 2022)). These different resistance-conferring genes can be “pyramided” to ensure broad and durable resistance to *G. pallida.*

We have identified a new source of resistance to *G. pallida* mapping to chromosome VI in the wild potato species accession *S.* *spegazzinii* CPC 7195, which we refer to as Gpa VI^l^_spg_. This accession also shows an independent resistance to *G.* *rostochiensis,* although no further characterisation of this source has been performed to date. Both a KASP assay specific to the *H1* resistance (Vanessa Young, personal communication) and dRenSeq analysis using recently established candidates for *H1* (Ingo Hein, personal communication) showed that the *H1* resistance is not present in *S.* *spegazzinii* CPC 7195, but it remains to be determined if this accession contains the *Gro1* gene (Ramakrishnan et al. 2015). Our data show that the *G. pallida* and *G.* *rostochiensis* resistances segregate independently and are therefore not conferred by a single locus. Resistances to both *G. pallida* and *G.* *rostochiensis* have been described previously in other accessions of the wild potato species *S.* *spegazzinii* Bitter (*e.g*. Kreike et al. 1994) notably GpaM2 on chromosome VI, which only has a small effect on the resistance to *G. pallida* and is located near QTLs which confer resistance to *P.* *infestans* (Caromel et al. 2003).

For *G. pallida*, the new resistance from *S.* *spegazzinii* accession CPC 7195 reduces the number of female nematodes by more than 95% for three populations representing the different introductions present in the UK. This makes this resistance a desirable target for introgression into commercial potato cultivars. However, for the resistance described here on chromosome VI, this remains to be proven, as only one population (Lindley, Pa2/3) was used for mapping. For *G.* *rostochiensis* Ro1, the reduction in female numbers is slightly less, although it is more than 90% in *S.* *spegazzinii* accession CPC7195.

### Mapping the *G. pallida* resistance locus derived from *S. spegazzinii* CPC 7195

Two different genotyping platforms (GenSeq and GBS) provided informative SNPs that showed linkage to the resistance vs susceptibility phenotype. The KASP assays derived from SNPs obtained from data of GenSeq and GBS both support the location of a major resistance locus on chromosome VI. Furthermore, the two plants in the resistant bulk chosen for enrichment sequencing that had been identified by KASP markers as susceptible had their phenotype re-confirmed, which also supports this result. As a result, the resistance locus could be mapped with high certainty to a region on chromosome VI, between positions 56,795,582 and 56,9145,572 that corresponds to an interval of 118 kb based on the DM genome assembly containing 14 high-confidence gene models in the reference genome of DM (v6.01). Interestingly, none of the genes identified in the region are NB-LRR genes. However, this is based on the DM reference genome and not the species harbouring the resistance, *S.* *spegazzinii,* which is taxonomically quite distinct from *S. tuberosum* Group Phureja. There is, as yet, no genome sequence for *S.* *spegazzinii* so it remains to be seen if the resistance gene mapped to linkage group VI is an NB-LRR gene. So far, there is no indication that *R*-genes are present in the region containing the resistance locus determined by GenSeq. However, there is a possibility that in *S.* *spegazzinii*, a gene conversion event or translocation of *R*-genes has occurred since divergence of this species from landraces such as *S.* *tuberosum* Group Phureja. Such phenomena are not uncommon and have been shown for example for *Mi-*1 gene homologues in *Solanum* species (Sanchez-Puerta and Masuelli 2011). If this were the case, the distance of the flanking markers would be larger than in DM*.* Although NB-LRR genes are an important family conferring resistance to diseases and pests, *R*-protein independent mechanisms for resistance have been described. One example is the resistance to the soybean cyst nematode *Heterodera glycines*. Two loci, *rhg1* and *rhg4*, confer resistance independently. Protein products from the allele rhg1b from soybean accession PI 88788 are cytotoxic and are accumulated in the nematode feeding site, thereby conferring full resistance to *H.* *glycines*, whereby the level of resistance is dependent on the copy number of the genes on locus *rhg1* (Cook et al. 2012). This resistance is broadly used in the USA. Another resistance, from a Peking accession, requires low copy numbers of the allele *rhg1a* and the resistance allele of the dominant quantitative trait locus (QTL) *Rhg4* (Shaibu et al. 2020). A serine hydroxy methyl transferase has been identified as responsible for the resistance to *H.* *glycines* (reviewed in Siddique and Jones 2021). For resistance to PCN, no similar mechanism is known yet, however (Butler et al. 2019) showed that potatoes transformed with the soybean *Rhg1* locus become resistant to PCN. Most plants synthesise toxic compounds, either constitutively or in response to attacks by pathogens or herbivores, that might provide a more general protective effect against pathogens and pests than resistance derived from *R*-genes. Wolters et al. 2023 identified two glycosyltransferase genes that provide resistance to early blight caused by *Alternaria solani* in the potato species *S.* *commersonii* and *S.* *malmeanum* by BSA on 37 genotypes. However, a generic toxic response in *S.* *spegazzinii* is unlikely, as such a response would most likely be effective against both *G. pallida* and *G.* *rostochienis*. At the current time, we cannot determine whether the newly identified *G. pallida* resistance described here is due to an *R*-gene or a different resistance mechanism. In summary, we have utilised two highly complementary genetic tools, GenSeq and GBS in combination with PCR-based targeted sequencing to independently verify the main resistance locus on potato chromosome VI. SNPs that were identified by all approaches were successfully converted into KASPs and delimited the interval to 118 kb using DM as a reference.

### Supplementary Information

Below is the link to the electronic supplementary material.

Supplementary file1 (DOCX 47 kb)

## Data Availability

GenSeq and GBS sequence data have been deposited in the European Nucleotide Archive (ENA) at EMBL-EBI (https://www.ebi.ac.uk/ena) under accession numbers PRJEB73769 and PRJEB158732.

## Footnotes

1 http://spuddb.uga.edu/jbrowse/index.html?data=data%2FDM_potato_v61&loc=chr06%3A56796628..56914618&tracks=DNA%2Cloci%2Chc_gene_models&highlight= = 
