# Supplementary material for: Characterisation and mapping of a Globodera pallida resistance derived from the wild potato species Solanum spegazzinii
Source: Theor Appl Genet. 2024 Apr 16;137(5):106. doi: 10.1007/s00122-024-04605-0 (PMC11018675; doi:10.1007/s00122-024-04605-0)
Supplement: Supplementary file 1 — Supplementary file1 (DOCX 47 kb) [file 122_2024_4605_MOESM1_ESM.docx]

**Supplementary Table S1: Informative SNP obtained by GenSeq and GBS that were used to design KASP and sequencing assays**

The genomic sequence of potato genotype Desirée version DM4.03 was used as reference genome.

| **Assay**  **type** | **Position**  **on**  **chr. VI** | **Parents’**  **Alleles**  **Res. Susc.** | | **Primer sequence allele X (FAM)** | **Primer sequence allele Y (HEX)** | **Primer sequence common** | |
| --- | --- | --- | --- | --- | --- | --- | --- |
| GenSeq | 53821580 | A/T | A | GCGAGTTTCCTCGTCTGCTTCA | GCGAGTTTCCTCGTCTGCTTCT | | CCTTCGATACTCTCGAACAACACTTAATT |
| GenSeq | 54218046 | T/C | C | CCATTGGAACATATGCTTCATACTGT | CCATTGGAACATATGCTTCATACTGC | | CAACGCGTGATACATTTGATGGTAAAGAA |
| GenSeq | 55096874 | C/T | T | AAACGATCCCAACCATTCCTTTGTC | GAAACGATCCCAACCATTCCTTTGTT | | CGCGATGATGGGACACTGTCTAAAT |
| GenSeq | 55909792 | G/C | G | CCCTATTTTACTAGCTATCTCAAAACAG | CCCTATTTTACTAGCTATCTCAAAACAC | | ACCTTCGGGCATTGGGAACACATT |
| GBS | 56135692 | C/T | T | GACCATTTGTTTGGTATGTTATAATTGCC | AGACCATTTGTTTGGTATGTTATAATTGCT | | GATGGAGGAAGCTAARATTGGAATAAAGTA |
| GBS | 56915767 | T/G | T | GAAGTGAATCAATACATTGCCAGAAT | GAAGTGAATCAATACATTGCCAGAAG | | CACCTGGGCTCCCTCTGGAT |
| GBS | 56987876 | T/G | T | AACCAACTTGACGTGAGCAGAAGA | CCAACTTGACGTGAGCAGAAGC | | GAGGTTAACGAAGGCAAGTATATGTCAAA |
| GenSeq | 57322514 | G/A | G | CCTTGACATGAGGATCTGTGTAC | AGCCTTGACATGAGGATCTGTGTAT | | CATCTGCGTTTCTCTTTTGAGAATCCAAA |
| GenSeq | 58781798 | G/A | G | AATGCAAATGCAAGAAACGAAGGTAG | GAATGCAAATGCAAGAAACGAAGGTAA | | CCTGAGTGTCTCTTCCAAAGTGTRAAA |
| **Assay type** | **Position**  **on** | **Parent’s**  **Alleles** | | **Primer sequence (5'->3’)** | |  |  |
|  | **chr. VI** | **Res.** | **Susc.** | **Forward primer** | **Reverse primer** |  |  |
| GBS | 56796582 | C/T | C | GTTTTCAGAGCGAGCACAC | AGCTTCTCAACAGCTTGAAC |  |  |
| GBS | 56914572 | A/G | G | GTATCACAAGGGGTCAAACATC | GAAGACCATTCTTCACAGCTAC |  |  |

**Supplementary Table S2 Number of female nematodes for selected 13.A.02 progeny and parents**

Panel A shows the clone IDs that were used for the bulk segregation analysis, the average nematode number of 3 or 4 independent replicates per clone and the standard deviation. Note that clones 13.A.02(619) and 13.A.02(625) were re-phenotyped in a pot bio-assay, for details see main text. Panel B shows the clone IDs of the recombinant plants used for the mapping of the resistance locus with their number of female nematodes and standard deviation obtained from a root trainer assay.

**A**

|  | **Susceptible plants** | | | **Resistant plants** | | |
| --- | --- | --- | --- | --- | --- | --- |
|  | **Clone ID** | **Number of**  **female nematodes** | **Standard**  **deviation** | **Clone ID** | **Number of**  **female nematodes** | **Standard**  **deviation** |
| **Bulk** | 13.A.02(58) | 84 | 31.1 | 13.A.02(49) | 2 | 1.2 |
|  | 13.A.02(444) | 77 | 35.3 | 13.A.02(70) | 1 | 1.7 |
|  | 13.A.02(448) | 74 | 14.4 | 13.A.02(446) | 1 | 0.8 |
|  | 13.A.02(465) | 109 | 56.9 | 13.A.02(463) | 0 | 0.4 |
|  | 13.A.02(467) | 75 | 34.9 | 13.A.02(469) | 1 | 0.8 |
|  | 13.A.02(471) | 99 | 51.0 | 13.A.02(495) | 1 | 0.8 |
|  | 13.A.02(479) | 125 | 40.0 | 13.A.02(500) | 1 | 0.7 |
|  | 13.A.02(510) | 78 | 81.3 | 13.A.02(512) | 1 | 0.9 |
|  | 13.A.02(519) | 110 | 29.0 | 13.A.02(516) | 1 | 0.9 |
|  | 13.A.02(524) | 75 | 23.3 | 13.A.02(523) | 1 | 0.5 |
|  | 13.A.02(545) | 95 | 43.1 | 13.A.02(568) | 1 | 0.8 |
|  | 13.A.02(566) | 87 | 23.8 | 13.A.02(579) | 0 | 0.4 |
|  | 13.A.02(608) | 82 | 56.4 | 13.A.02(586) | 1 | 1.2 |
|  | 13.A.02(615) | 99 | 35.1 | 13.A.02(619) | 1 | 0.9 |
|  | 13.A.02(628) | 108 | 31.2 | 13.A.02(625) | 2 | 0.8 |
|  | 13.A.02(635) | 97 | 26.9 | 13.A.02(633) | 1 | 0.8 |
|  | 13.A.02(637) | 102 | 29.0 | 13.A.02(648) | 1 | 1.2 |
|  | 13.A.02(638) | 101 | 60.2 | 13.A.02(678) | 1 | 0.9 |
|  | 13.A.02(643) | 89 | 23.7 | 13.A.02(686) | 1 | 0.8 |
|  | 13.A.02(644) | 117 | 30.1 | 13.A.02(700) | 1 | 1.1 |
| **Parent** |  |  |  |  |  |  |
|  | DB337(37) | 122 | 15.2 | 03.F1.3a(35) | 1 | 1.2 |

|  |  |  |  |  |  | |  |
| --- | --- | --- | --- | --- | --- | --- | --- |
| **B** | **Susceptible plants** | | | **Resistant plants** | | | |
|  | **Clone ID** | **Number of**  **female nematodes** | **Standard**  **deviation** | **Clone ID** | **Number of**  **female nematodes** | | **Standard**  **deviation** |
|  | 13.A.02(22) | 37 | 7.1 | 13.A.02(33) | 2 | | 0.9 |
|  | 13.A.02(128) | 29 | 8.6 | 13.A.02(136) | 4 | | 1.5 |
|  | 13.A.02(188) | 43 | 10.4 | 13.A.02(290) | 5 | | 0.4 |
|  | 13.A.02(251) | 36 | 7.3 | 13.A.02(398) | 2 | | 0 |
|  | 13.A.02(265) | 59 | 20.5 | 13.A.02(419) | 5 | | 1.3 |
|  | 13.A.02(292) | 67 | 29.7 | 13.A.02(435) | 2 | | 1.1 |
|  | 13.A.02(361) | 51 | 14.1 | 13.A.02(441) | 3 | | 1.7 |
|  | 13.A.02(373) | 51 | 15.1 | 13.A.02(456) | 1 | | 0.3 |
|  | 13.A.02(393) | 64 | 12.0 | 13.A.02(457) | 4 | | 2.3 |
|  | 13.A.02(422) | 31 | 5.0 | 13.A.02(554) | 3 | | 1.1 |
|  | 13.A.02(524) | 26 | 1.7 | 13.A.02(569) | 5 | | 2.7 |
|  | 13.A.02(537) | 30 | 3.5 | 13.A.02(579) | 3 | | 1.1 |
|  | 13.A.02(543) | 31 | 11.3 | 13.A.02(640) | 0 | | 0.0 |
|  | 13.A.02(558) | 30 | 9.1 | 13.A.02(641) | 1 | | 0.4 |
|  | 13.A.02(615) | 48 | 12.4 | 13.A.02(674) | 3 | 1.1 | |
|  | 13.A.02(652) | 29 | 4.0 | 13.A.02(679) | 2 | | 0.9 |
|  | 13.A.02(702) | 55 | 43.1 | 13.A.02(686) | 5 | | 0.3 |
|  | 13.A.02(757) | 60 | 23.8 | 13.A.02(723) | 1 | | 0.8 |
|  | 13.A.02(787) | 61 | 29.6 | 13.A.02(783) | 2 | | 0.9 |
|  | 13.A.02(803) | 26 | 5.8 | 13.A.02(787) | 4 | | 1.8 |
|  | 13.A.02(815) | 27 | 6.6 | 13.A.02(944) | 4 | | 2.1 |
|  | 13.A.02(873) | 27 | 5.8 | 13.A.02(955) | 1 | | 0.4 |
|  | 13.A.02(874) | 48 | 11.3 |  |  | |  |
|  | 13.A.02(919) | 37 | 11.3 |  |  | |  |
|  | 13.A.02(925) | 39 | 10.2 |  |  | |  |
|  | 13.A.02(985) | 38 | 2.4 |  |  | |  |
| **Parent** |  |  |  |  |  | |  |
|  | DB337(37) | 49 | 9.6 | 03.F1.3a(35) | 2 | | 1.7 |

**Supplementary Table S3 Read numbers and on-target reads to the reference DM in the GenSeq experiment**

| **Library** | **Paired end**  **reads** | **On target mapping reads (%) to reference DM at mismatch rate** | | | |
| --- | --- | --- | --- | --- | --- |
|  |  | **2%** | **3%** | **5%** | **10%** |
| **Susc. parent** | 1084865 | 758946 (70.0) | 831000 (76.6) | 903072 (83.2) | 9693835 (88,8) |
| **Res. parent** | 1246752 | 657153 (52.8) | 776376 (62.7) | 922222 (74.0) | 1065983 (85.5) |
| **Susc. bulk** | 1508457 | 909928 (60.3) | 1032420 (68.4 | 1172930 (77.8) | 1306989 (86.4) |
| **Res. bulk** | 2263405 | 1367635 (60.4) | 1552759 (68.6) | 1763464 (86.7) | 1961416 (86.7) |

**Supplementary Table S4 Different mismatch rates provided different number of SNPs in GenSeq**

|  | **Number of SNPs (intragenic)** | | | |  | |  |
| --- | --- | --- | --- | --- | --- | --- | --- |
| **Mismatch rate** | **Bulks** | **Parents** | **Bulks & parents *** | **Location of informative SNPs** | |  |  |
| **2%** | 41 | 1794 | 15 | 12 on chr. VI and 2 on chr. XII | |  |  |
| **3%** | 48 | 3112 | 14 | 11 on chr. VI and 1 each on chr. IX, X, and XII | | | |
| **5%** | 77 | 4637 | 24 | 20 on chr. VI, 3 on chr. IX, and 1 on XII | | | |
| **10%** | 79 | 5717 | 22 | 20 on chr. VI and 1 each on chr. IX, and XII | | | |

* SNPs that are present in both bulks and parents, informative SNPs

**Supplementary Table S5 Informative SNP candidates detected from the GBS experiment**

Three SNPs were used to create KASP assays: their primer sequences are shown in Supplementary Table S4 section B. Sequencing primers were designed for the other SNPs.

| **Mismatch**  **rate** | **Chr.** | **Start** | **Stop** | **Gene ID** | **No. of**  **SNPs** | | **SNP Position (Alleles)** |
| --- | --- | --- | --- | --- | --- | --- | --- |
| **2%** | VI | 53821292 | 53822172 | PGSC0003DMG400027013 | 1 | | 53821580 (A/T) |
|  | VI | 54660358 | 54663473 | PGSC0003DMG400005895 | 1 | | 54661772 (G/A) |
|  | VI | 55904773 | 55910691 | PGSC0003DMG400007504 | 6 | | 55909792 (G/C), 55910203 (T/C), 55910206 (A/C), 55910257 (C/G), 55910404 (C/G), 55910409 (G/C) |
|  | VI | 57309861 | 57313784 | PGSC0003DMG400030365 | 1 | | 57313272 (G/A) |
|  | VI | 57314827 | 57323565 | PGSC0003DMG400030366 | 2 | | 57322514 (G/A), 57322806 (A/T) |
|  | VI | 58776636 | 58782360 | PGSC0003DMG400020145 | 1 | | 58781798 (G/A) |
|  | IX | 54146053 | 54150042 | PGSC0003DMG400032241 | 1 | | 54146755 (G/A) |
|  | XII | 21536758 | 21537672 | PGSC0003DMG400014943 | 1 | | 21536868 (T/A) |
|  | XII | 49744644 | 49746244 | PGSC0003DMG400028627 | 1 | | 49746075 (C/T) |
| **3%** | VI | 53821292 | 53822172 | PGSC0003DMG400027013 | 1 | | 53821580 (A/T) |
|  | VI | 54660358 | 54663473 | PGSC0003DMG400005895 | 1 | | 54661772 (G/A) |
|  | VI | 55904773 | 55910691 | PGSC0003DMG400007504 | 6 | | 55909792 (G/C), 55910203 (T/C), 55910206 (A/C), 55910257 (C/G), 55910404 (C/G), 55910409 (G/C) |
|  | VI | 57309861 | 57313784 | PGSC0003DMG400030365 | 1 | | 57313272 (G/A) |
|  | VI | 57314827 | 57323565 | PGSC0003DMG400030366 | 2 | | 57322514 (G/A), 57322806 (A/T) |
|  | IX | 54146053 | 54150042 | PGSC0003DMG400032241 | 1 | | 54146755 (G/A) |
|  | X | 28756497 | 28758184 | PGSC0003DMG400017694 | 1 | | 28757235 (T/C) |
|  | XII | 49744644 | 49746244 | PGSC0003DMG400028627 | 1 | | 49746075 (C/T) |
| **5%** | VI | 46214746 | 46219803 | PGSC0003DMG400026085 | 1 | | 46215098 (A/G) |
|  | VI | 53821292 | 53822172 | PGSC0003DMG400027013 | 1 | | 53821580 (A/T) |
|  | VI | 54217175 | 54219255 | PGSC0003DMG400005919 | 1 | | 54218046 (T/C) |
|  | VI | 54660358 | 54663473 | PGSC0003DMG400005895 | 1 | | 54661772 (G/A) |
|  | VI | 55096366 | 55102143 | PGSC0003DMG400007149 | 3 | | 55096799 (C/T), 55096832 (A/G), 55096874 (C/T) |
| Supplementary Table S5 continued | | | |  |  |  | |
| **Mismatch**  **rate** | **Chr.** | **Start** | **Stop** | **Gene ID** | **No. of**  **SNPs** | | **SNP Position (Alleles)** |
| **5%** | VI | 55904773 | 55910691 | PGSC0003DMG400007504 | 6 | | 55909792 (G/C), 55910203 (T/C), 55910206 (A/C), 55910257 (C/G), 55910404 (C/G), 55910409 (G/C) |
|  | VI | 57309861 | 57313784 | PGSC0003DMG400030365 | 1 | | 57313272 (G/A) |
|  | VI | 57314827 | 57323565 | PGSC0003DMG400030366 | 6 | | 57322514 (G/A), 57322806 (A/T), 57323327 (C/G), 57323385 (T/C), 57323393 (A/T), 57323514 (A/G) |
|  | IX | 53672502 | 53677067 | PGSC0003DMG400030078 | 2 | | 53673705 (A/C), 53673779 (G/C) |
|  | IX | 54146053 | 54150042 | PGSC0003DMG400032241 | 1 | | 54146755 (G/A) |
|  | XII | 49744644 | 49746244 | PGSC0003DMG400028627 | 1 | | 49746075 (C/T) |
| **10%** | VI | 52509378 | 52510568 | PGSC0003DMG400026990 | 1 | | 52510349 (C/T) |
|  | VI | 53821292 | 53822172 | PGSC0003DMG400027013 | 1 | | 53821580 (A/T) |
|  | VI | 54217175 | 54219255 | PGSC0003DMG400005919 | 1 | | 54218046 (T/C) |
|  | VI | 54660358 | 54663473 | PGSC0003DMG400005895 | 1 | | 54661772 (G/A) |
|  | VI | 55096366 | 55102143 | PGSC0003DMG400007149 | 4 | | 55096794 (G/C), 55096799 (C/T), 55096832 (A/G), 55096874 (C/T) |
|  | VI | 55904773 | 55910691 | PGSC0003DMG400007504 | 6 | | 55909792 (G/C), 55910203 (T/C), 55910206 (A/C), 55910257 (C/G), 55910404 (C/G), 55910409 (G/C) |
|  | VI | 57309861 | 57313784 | PGSC0003DMG400030365 | 1 | | 57313272 (G/A) |
|  | VI | 57314827 | 57323565 | PGSC0003DMG400030366 | 5 | | 57322514 (G/A), 57322806 (A/T), 57323385 (T/C), 57323393 (A/T), 57323514 (A/G) |
|  | IX | 54146053 | 54150042 | PGSC0003DMG400032241 | 1 | | 54146755 (G/A) |
|  | XII | 49744644 | 49746244 | PGSC0003DMG400028627 | 1 | | 49746075 (C/T) |
